# The spectrum of retinopathy in adults with *Plasmodium falciparum* malaria

**DOI:** 10.1016/j.trstmh.2009.03.001

**Published:** 2009-07

**Authors:** Richard J. Maude, Nicholas A.V. Beare, Abdullah Abu Sayeed, Christina C. Chang, Prakaykaew Charunwatthana, M. Abul Faiz, Amir Hossain, Emran Bin Yunus, M. Gofranul Hoque, Mahtab Uddin Hasan, Nicholas J. White, Nicholas P.J. Day, Arjen M. Dondorp

**Affiliations:** a Mahidol–Oxford Tropical Medicine Research Unit, Faculty of Tropical Medicine, Mahidol University, 420/6 Rajvithi Road, Bangkok 10400, Thailand; b Centre for Clinical Vaccinology and Tropical Medicine, Nuffield Department of Clinical Medicine, John Radcliffe Hospital, Oxford OX3 7LJ, UK; c St Paul's Eye Unit, Royal Liverpool University Hospital, Prescot Street, Liverpool L7 8XP, UK; d Chittagong Medical College Hospital, Chittagong, Bangladesh; e Malaria Research Group (MRG), 1051/A, O.R Nizam Road, Mehdibag, Chittagong, Bangladesh

**Keywords:** Cerebral malaria, *Plasmodium falciparum*, Retinopathy, Pathophysiology, Microcirculation, Bangladesh

## Abstract

A specific retinopathy has been described in African children with cerebral malaria, but in adults this has not been extensively studied. Since the structure and function of the retinal vasculature greatly resembles the cerebral vasculature, study of retinal changes can reveal insights into the pathophysiology of cerebral malaria. A detailed observational study of malarial retinopathy in Bangladeshi adults was performed using high-definition portable retinal photography. Retinopathy was present in 17/27 adults (63%) with severe malaria and 14/20 adults (70%) with cerebral malaria. Moderate or severe retinopathy was more frequent in cerebral malaria (11/20, 55%) than in uncomplicated malaria (3/15, 20%; *P* = 0.039), bacterial sepsis (0/5, 0%; *P* = 0.038) or healthy controls (0/18, 0%; *P* < 0.001). The spectrum of malarial retinopathy was similar to that previously described in African children, but no vessel discolouration was observed. The severity of retinal whitening correlated with admission venous plasma lactate (*P* = 0.046), suggesting that retinal ischaemia represents systemic ischaemia. In conclusion, retinal changes related to microvascular obstruction were common in adults with severe falciparum malaria and correlated with disease severity and coma, suggesting that a compromised microcirculation has important pathophysiological significance in severe and cerebral malaria. Portable retinal photography has potential as a valuable tool to study malarial retinopathy.

## 1 Introduction

Every year more than one million people die from severe malaria.^1^ Cerebral malaria with coma is one of the most important manifestations of adult severe malaria, however its pathophysiology is still incompletely understood and this has hindered the development of more effective therapies.^2^ Because of the similarity of the retinal and cerebral microvasculatures, the directly accessible retinal circulation has been studied as a surrogate for the cerebral circulation in order to investigate cerebral malaria.^3^

In recent years, a unique spectrum of retinal changes has been described in African children with severe malaria, including retinal whitening, haemorrhages, vessel discolouration and in some cases papilloedema.^4^, ^5^ Using indirect ophthalmoscopy, retinopathy can be seen in approximately two-thirds of paediatric patients with cerebral malaria and the severity of retinopathy correlates with the severity of malaria, mortality and duration of coma.^4^, ^6^ Currently there is debate whether the pathophysiological mechanisms leading to cerebral and severe malaria differ between adults and children.^7^ In adult severe and cerebral malaria, retinal haemorrhages have also been described^8^ but other features of malarial retinopathy requiring indirect ophthalmoscopy or other techniques have been studied less extensively, which prompted the current study. The specific ophthalmoscopic technique used is important because malaria-specific retinal whitening and vessel discolouration can be more prominent in the peripheral retina and thus may not be identified in the field by a non-expert using a direct ophthalmoscope.^5^ With indirect ophthalmoscopy there is high interobserver concordance for grading the severity of findings, including retinal whitening.^9^

Retinal photography has a number of advantages over direct and indirect ophthalmoscopy for assessing malarial retinopathy. As images are recorded for later examination, each can be carefully scrutinised by multiple observers for much longer than would be possible with ophthalmoscopy; objectivity is therefore higher. The greater magnification and high resolution of modern fundus cameras and the possibility of adjusting colour and contrast with imaging software also allow subtle details to be detected more easily, which increases sensitivity. Currently, portable retinal cameras are available that can be used at the bedside, even in the sickest patients.

An observational study was conducted using bedside portable high-resolution digital retinal photography in adults with severe and uncomplicated malaria compared with patients with sepsis and healthy volunteers in order to establish the full spectrum of malarial retinopathy.

## 2 Materials and methods

### 2.1 Study site and patients

This study was conducted at Chittagong Medical College Hospital, a large 1000-bed teaching hospital in Chittagong, Bangladesh, from June–August 2008. Malaria transmission is seasonal and of low intensity in this location.

Consecutive adult patients (>16 years) with slide-confirmed severe or uncomplicated falciparum malaria, according to modified WHO criteria,^10^ were recruited if written informed consent was obtained from the attending relative. Two control groups were also studied: healthy relatives of enrolled patients and randomly selected patients with sepsis.^11^ Healthy relatives were recruited for comparison with the background prevalence of retinal changes in the healthy population. Exclusion criteria were: patients unable or unwilling to co-operate with eye examination; contraindications to tropicamide eye drops, such as angle closure glaucoma or documented allergy; and patients with severe corneal scarring or cataracts in both eyes precluding ophthalmoscopy and retinal photography.

### 2.2 Study procedures

On admission, a full history and examination were carried out. Blood samples were obtained for haemoglobin, haematocrit, parasitaemia, platelet count, white cell count, plasma lactate levels, glucose levels and full biochemistry. Eye examination included pupillary reaction to light and accommodation and direct and indirect ophthalmoscopy. Ophthalmoscopy was done within 30 min of administration of two drops of 0.5% or 1% tropicamide. In addition, all patients had digital photographs taken of both retinas whenever possible. To obtain images including the peripheral retina, a minimum of nine overlapping photographs (corresponding to approximately 15 megapixels) were required from each retina. These photographs were then amalgamated and merged using Photoshop CS3 Extended software (Adobe, San José, CA, USA). Photographs were examined by a blinded investigator for evidence of malarial or other retinopathy, and changes were graded as mild, moderate or severe according to the classification of Beare et al.^9^ and Harding et al.^12^ Grading is determined by: the size of the affected area of retinal whitening relative to the optic disk; number of retinal haemorrhages and proportion of these haemorrhages that are white-centered; the extent of vessel discolouration; and the severity of papilloedema. Fifteen percent of the photographs were randomly selected and sent to a second, expert, blinded investigator for grading as a check of concordance.

### 2.3 Drug and supportive treatments

Antimalarial drug treatment was with i.v. artesunate (Guilin Pharmaceutical Factory, Guangxi, People's Republic of China) at 2.4 mg/kg body weight on admission followed by 2.4 mg/kg at 12 h and 24 h and then every 24 h. When the patient was able to take food, treatment was switched to complete a standard six-dose course of oral artemether plus lumefantrine for a further 3 days. Supportive treatments were in accordance with the 2006 WHO guidelines^13^ and local hospital guidelines, but the availability of renal replacement therapy and mechanical ventilation was limited.

### 2.4 Statistical analysis

Analysis was performed using Excel 2007 (Microsoft Corp., Redmond, WA, USA), SPSS version 15.0 (SPSS Inc., Chicago, IL, USA) and Stata SE 10 (Stata Corp., College Station, TX, USA). Correlations were assessed using Spearman's rank method for non-parametric data. Fisher's exact test was used to compare retinal severity scores between groups. The trend of increasing severity of retinopathy with increasing malaria severity was assessed by *P* for trend. The level of significance was *P* < 0.05.

## 3 Results

A total of 66 patients were enrolled. Of these, 42 patients had falciparum malaria, of whom 27 had severe malaria, including 20 with coma [Glasgow Coma Scale (GCS) score <11)], and 15 had uncomplicated malaria. In addition, 19 healthy volunteers and 5 patients with sepsis were recruited. One of the healthy volunteers appeared to have diabetes with associated retinopathy and was therefore excluded from further analysis. Nine patients died, all of whom had severe malaria. The mean ages of patients were similar in all groups: severe malaria 38.1 years (range 17–75 years); uncomplicated malaria 33.0 years (range 17–63 years); healthy controls 34.9 years (range 19–49 years); and sepsis controls 25.0 years (range 17–40 years). The number of males in each group was 21/27 (77.8%), 11/15 (73.3%), 14/18 (77.8%) and 1/5 (20.0%), respectively. The distribution of presenting severity symptoms in patients with severe malaria is shown in Table 1. All patients with uncomplicated malaria presented with fever and flu-like symptoms, 11 had vomiting and 1 had diarrhoea. One of the patients with sepsis was unconscious (GCS score = 3/15) with suspected bacterial meningitis on admission, one had bronchopneumonia and the other three had sepsis of unknown origin.

**Table 1:** Distribution of presenting severity symptoms in patients with severe malaria (patients with uncomplicated malaria had none of these features)

Symptom	No. (%) of patients (*n* = 27)
GCS score <11	20 (74)
Haematocrit <20% with parasite count >100 000/mm^3^	3 (11)
Bilirubin >3.0 mg/dl with parasite count >100 000/mm^3^	15 (56)
Serum creatinine >3.0 mg/dl	9 (33)
Systolic blood pressure <80 mmHg with cool extremities	1 (4)
Peripheral asexual stage parasitaemia >5%	5 (19)
Venous lactate >4 mmol/l	12 (44)
Venous bicarbonate <15 mmol/l	11 (41)

GCS: Glasgow Coma Scale.

A mean of 11 (95% CI 9–14) usable photos were taken of each retina. For three patients with cerebral malaria and three patients with non-cerebral malaria, the photographs were judged to be insufficiently clear to exclude mild retinopathy, although their retinas appeared normal by indirect ophthalmoscopy. This loss of clarity was caused by rapid eye movements, which also made it difficult to perform adequate indirect ophthalmoscopy. In addition, two of the patients with severe malaria had severe corneal scarring in one eye so only the opposite retina could be examined.

The technique of portable retinal photography was found to be highly practical in all groups of patients in this study. Clear images could also be obtained from patients with involuntary wandering eye movements often found in patients with cerebral malaria. Concordance of overall grading of retinopathy between the two blinded investigators was 100%.

### 3.1 Retinal findings

**Figure 1:**
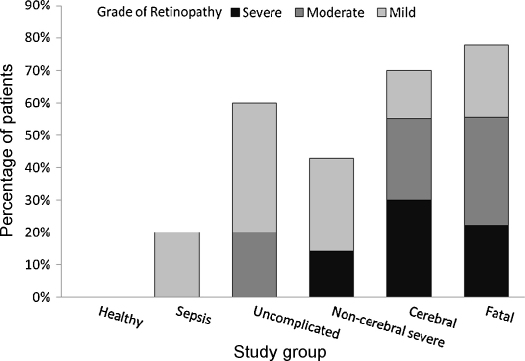
Severity of retinal changes consistent with malarial retinopathy in patients with *Plasmodium falciparum* malaria or sepsis and healthy volunteers.

Features compatible with malarial retinopathy were present in 17/27 patients (63%) with severe malaria, 7/9 patients (78%) with a fatal course, 14/20 patients (70%) with cerebral malaria, 3/7 patients (43%) with non-cerebral severe malaria, 9/15 patients (60%) with uncomplicated malaria, 1/5 patients (20%) with sepsis and 0/18 (0%) of the ‘healthy’ volunteers (Figure 1). There was a significantly higher proportion with retinopathy in all those with malaria than in healthy controls (*P* < 0.001). The severity of retinal findings correlated with severity of disease categorised from ‘healthy’ volunteers, uncomplicated, non-fatal non-cerebral severe, non-fatal cerebral to fatal malaria. This was true for the total retinopathy severity score (*P* for trend = 0.001), number of retinal haemorrhages (*P* for trend = 0.001) and severity of retinal whitening (*P* for trend = 0.001). The sensitivities and specificities of retinopathy for detecting malaria are shown in Table 2. One patient with sepsis had a single cotton wool spot on the fovea and was classed as mild retinopathy although they did not have any of the retinal features thought to be specific to malaria. None of the patients with malaria or sepsis had diabetes or hypertension.

**Table 2:** Sensitivity and specificity of any retinopathy and moderate-to-severe retinopathy for malaria of different severities

Severity	Any retinopathy		Moderate-severe retinopathy	
	Sensitivity (%)	Specificity (%)	Sensitivity (%)	Specificity (%)
All malaria	62	92	36	100
Severe	63	72	44	92
Cerebral	70	70	55	91
Fatal	78	63	56	89

Moderate or severe retinopathy was present in 12/27 patients (44%) with severe malaria, 5/9 patients (56%) with fatal malaria, 11/20 patients (55%) with cerebral malaria, 1/7 patients (14%) with non-cerebral severe malaria, 3/15 patients (20%) with uncomplicated malaria, 0/5 patients (0%) with sepsis and 0/18 healthy volunteers (0%) (Figure 1). Sensitivities and specificities are shown in Table 2. There was more moderate-to-severe retinopathy among those with cerebral malaria than uncomplicated malaria (*P* = 0.039), bacterial sepsis (*P* = 0.038) and healthy controls (*P* < 0.001). There were no significant differences in the prevalence or severity grade of retinopathy between fatal, cerebral and non-cerebral severe malaria.

**Figure 2:**
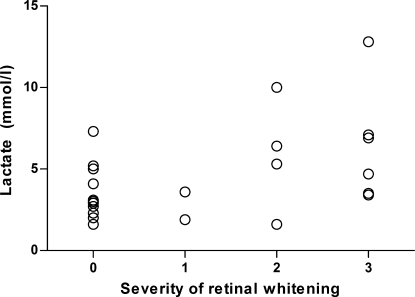
Scatter plot of serum lactate against severity of malarial retinopathy upon admission to hospital in 27 patients with severe malaria (*P* = 0.046).

The prevalences of individual features of retinopathy are shown in Table 3. The severity (i.e. number) of retinal haemorrhages correlated significantly with the severity of whitening (Spearman's *r* = 0.50; *P* = 0.009), and 16/21 patients (76%) with whitening also had haemorrhages. All patients with severe malaria who had whitening of the peripheral retina also had whitening of the macula. Admission venous plasma lactate levels in patients with severe malaria correlated significantly with the severity of retinal whitening (Spearman's *r* = 0.387; *P* = 0.046) (Figure 2) and the severity of macular whitening (Spearman's *r* = 0.387; *P* = 0.046). Haematocrit on admission correlated inversely with the number of white-centred haemorrhages (*P* = 0.013). There were no correlations between admission GCS score, haematocrit, parasitaemia, venous lactate, venous bicarbonate or the number of presenting clinical features of severe malaria (Table 1) and the severity of the different components of retinopathy. A summary of retinal findings is shown in Supplementary Table 1, including results from other studies for comparison.

**Table 3:** Prevalence of individual features of retinopathy in patients with *Plasmodium falciparum* malaria (vessel changes are not shown as none were seen in this study)

Group	Severity of retinopathy	Retinal findings [no. (%) of patients]							
		Any retinopathy	Haemorrhages		Papilloedema	Whitening			
			White-centred	Any		Macular	Foveal	Peripheral	Any
Cerebral (*n* = 20)	1	3 (15)	3 (15)	8 (40)	0	1 (5)	4 (20)	3 (15)	1 (5)
	2	5 (25)	3 (15)	3 (15)	0	4 (20)	2 (10)	2 (10)	4 (20)
	3	6 (30)	0	0	1 (5)	5 (25)	2 (10)	4 (20)	5 (25)
	All	14 (70)	6 (30)	11 (55)	1 (5)	10 (50)	8 (40)	9 (45)	10 (50)
Severe (*n* = 27)	1	5 (19)	3 (11)	10 (37)	0	3 (11)	6 (22)	5 (19)	3 (11)
	2	5 (19)	3 (11)	3 (11)	0	4 (15)	3 (11)	4 (15)	4 (15)
	3	7 (26)	1 (4)	1 (4)	2 (7)	6 (22)	2 (7)	4 (15)	6 (22)
	All	17 (63)	7 (26)	14 (52)	2 (7)	13 (48)	11 (41)	13 (48)	13 (48)
Uncomplicated (*n* = 15)	1	6 (40)	4 (27)	7 (47)	0	7 (47)	3 (20)	5 (33)	5 (33)
	2	3 (20)	0	0	0	1 (7)	0	3 (20)	3 (20)
	3	0	0	0	0	0	0	0	0
	All	9 (60)	4 (27)	7 (47)	0	8 (53)	3 (20)	8 (53)	8 (53)

**Figure 3:**
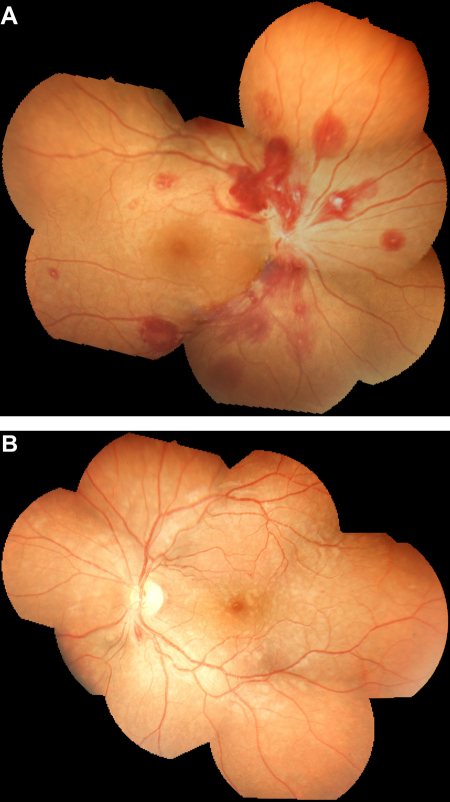
Examples of composite retinal photographs obtained from patients with severe malaria in this study. (A) Right retina of a 25-year-old woman with severe falciparum malaria manifest by profound anaemia and confusion [Glasgow Coma Scale (GCS) score = 14/15]. There are multiple white-centred haemorrhages and gross papilloedema, an unusual reported finding in adults with cerebral malaria. Parasitaemia was 80/1000 red cells, haematocrit was 8.2%, platelet count 86 × 10^3^/mm^3^ but coagulation parameters were normal. The patient received a blood transfusion but rapidly developed respiratory failure in the absence of chest signs. The patient died 12 h after admission before chest radiography or a cerebral CT scan could be performed. (B) Mosaic retinal whitening involving the entire macula and extensive areas of the peripheral retina in the left eye of a 24-year-old man with cerebral malaria (GCS score = 8/15), pulmonary oedema and Blackwater fever. Parasitaemia was 79/1000 red cells and haemoglobin 10.9 g/dl. The patient recovered consciousness within 48 h of starting i.v. artesunate and was discharged home after 6 days. Visual function and neurological examination were normal on discharge. The patients gave informed consent for their retinal photographs to be published.

Figure 3 shows two examples of composite retinal photographs of severe malarial retinopathy obtained with the portable digital retinal camera.

## 4 Discussion

This study demonstrates that the high prevalence and spectrum of ocular fundus findings in Bangladeshi adults with severe malaria is similar to that found previously in African children. This result differs from previous studies on malarial retinopathy in adults describing lower prevalences.^8^, ^14^, ^15^ In an Indian study, retinopathy was found in only 34.1% of 214 adults with cerebral malaria, and disk pallor was significantly associated with mortality.^14^, ^15^ In one Thai study of 150 adult and paediatric patients with cerebral malaria, 14.6% had retinal haemorrhages, and very few other retinal abnormalities were described.^8^ Both studies used predominantly direct ophthalmoscopy, precluding assessment of the peripheral retina,^5^ and were undertaken before malarial retinopathy in children was well described. In a detailed but small study using indirect ophthalmoscopy and fluorescein angiography, 8 (44%) of 18 adults with severe malaria had abnormal retinal findings. In this study, retinal capillary obstruction was present in five patients, three of whom had cerebral malaria, and demonstrated the association of capillary non-perfusion with cotton wool spots. Fluorescein leakage was associated with hyperlactataemia and renal impairment.^16^

There is currently debate whether the pathophysiology of severe and cerebral malaria differs between adults and children.^7^ Obstruction of microcirculatory blood flow by sequestered infected erythrocytes and other factors is thought to be a major contributor to the pathogenesis of acidosis, coma, death and neurological disability in both groups.^17^, ^18^ In addition, in children intravascular fibrin clots and accumulation of leukocytes and platelets have been described in fatal cases.^19^

In the current study some differences were noticed between the findings in adults compared with previous studies of children in Africa. The first is that blood vessel discolouration, found previously in approximately one-third of children with cerebral malaria,^4^ was not detected in any of the adults in this study. Vessel discolouration is thought to be due to dehaemoglobinisation of stationary erythrocytes infected with mature parasites in obstructed capillaries, arterioles and venules.^19^ It is unlikely that this was caused by the different technique since it has been shown previously that blood vessel discolouration in patients with severe malaria is seen easily on retinal photographs.^4^, ^5^, ^6^, ^19^ The second difference is that the prevalence of retinopathy in patients with uncomplicated malaria in this study was much higher than that found previously in children. As most of this retinopathy was mild, it is possible that this was due to the high sensitivity of the retinal photography used in this study. It should also be noted that a semi-immune patient presenting with uncomplicated falciparum malaria in an area of high transmission in Africa differs from a non-immune uncomplicated patient in a low transmission area in Bangladesh.

Although this was a small study, a number of interesting correlations emerged. In accordance with previous studies, there was a significant trend of increasing severity of retinopathy with increasing severity of malaria, and moderate-to-severe retinopathy was more common in severe and cerebral malaria than in uncomplicated malaria and the control groups. A planned larger study will enable a more detailed comparison between subgroups of patients.

Venous plasma lactate correlated with the severity of retinal whitening on admission. Lactate is a strong prognostic indicator in severe malaria and is caused by an increase in anaerobic glycolysis.^20^ Retinal whitening is due to obstruction of small blood vessels causing loss of retinal transparency through tissue hypoxia and localised retinal ischaemia[3], 21, 22 as shown by fluorescein angiographic studies.^23^ Our findings suggest that retinal whitening may not only reflect a compromised cerebral microvasculature associated with coma but also more general tissue ischaemia causing lactic acidosis and other vital organ dysfunction. Although lactic acidosis is also a prominent finding in severe sepsis, we did not find significant retinopathy in these patients, suggesting a different mechanism. The retinal microvascular obstruction in severe malaria is caused by sequestration of parasitised erythrocytes, but a role for microthrombus formation has also been suggested.^21^

The severity of retinal haemorrhages correlated with severity of malaria but also anaemia. This has been shown previously in adults with falciparum malaria.^14^, ^15^ Studies in children showed that the number of haemorrhages correlated with mortality^6^ and with the number of cerebral haemorrhages found post-mortem in those dying of cerebral malaria.^24^ Unlike retinal whitening and vessel discolouration, retinal haemorrhages do not coincide with occluded blood vessels on fluorescein angiography and a different pathological mechanism may be responsible, such as post-ischaemic endothelial cell damage.^25^

To our knowledge, this study is the first to examine malarial retinopathy systematically using high-resolution retinal photography. The methodology maximises objectivity and sensitivity in detecting retinal pathology. Owing to the underlying burden of corneal scarring and cataract disease, as well as involuntary eye movements typical of cerebral malaria, a small number of patients were difficult to examine and optimally clear retinal photographs were impossible to obtain. A limitation of this study was that many patients had limited tolerance to both retinal photography and indirect ophthalmoscopy when performed in quick succession, mainly due to the bright light involved in both techniques. For this reason, comprehensive indirect ophthalmoscopy was not performed in most non-comatose patients, excluding a comparative analysis between indirect ophthalmoscopy and retinal photography.

Paediatric severe malaria patients have never been studied in detail in Asia and comparison with findings in adults is important in the assessment of possible differences in pathophysiology between these groups. Retinal changes associated with microcirculatory obstruction can also be compared with changes in microcirculatory blood flow elsewhere in the body using orthogonal polarising spectroscopy.^26^ It is hoped that by assessing these multiple variables simultaneously, a clearer understanding of the role of microcirculatory obstruction in severe malaria will be obtained, which could have important consequences for the design of therapeutic interventions. Detailed assessment of retinopathy can also prove to be a valuable surrogate marker in intervention studies that aim to improve microcirculatory blood flow.^3^ A larger study of sufficient power will be required to examine the association of different presenting syndromes and disease outcome with retinopathy.

In conclusion, this study describes a new technique to document objectively and to describe the retinal changes associated with severe falciparum malaria. Malaria retinopathy is very common in adults with severe malaria, particularly cerebral malaria. The absence of retinal vessel changes in adults was conspicuous and may indicate a difference in the calibre of vessels involved in sequestration between adults and children. The presence of retinal whitening and its association with coma suggest that a compromised microcirculation has important pathophysiological significance in severe and cerebral malaria.

## Funding

The Mahidol–Oxford Tropical Medicine Research Unit is funded by the Wellcome Trust of Great Britain.

## Conflicts of interest

None declared.

## Ethical approval

Ethical clearance for the study was obtained from the Oxford Tropical Research Ethics Committee (OXTREC) and the Bangladesh Medical Research Council.

## Authors’ contributions

RJM, AMD, CCC and NAVB designed the study protocol; RJM, AAS, CCC and PC carried out the clinical assessments; MAF, AH, EBY, MGH and MUH helped with organisation and execution of the study; RJM carried out the analysis and interpretation of the data; RJM, AMD, NAVB, NJW, NPJD, AAS, MAF and CCC drafted the manuscript. All authors read and approved the final manuscript. AMD is guarantor of the paper.
