# Supplementary material for: The spectrum of retinopathy in adults with Plasmodium falciparum malaria
Source: Trans R Soc Trop Med Hyg. 2009 Jul;103(7):665–71. doi: 10.1016/j.trstmh.2009.03.001 (PMC2700877; doi:10.1016/j.trstmh.2009.03.001)
Supplement: Supplementary file 1 [file mmc1.doc]

**Supplementary Table 1 Percentage of patients with individual features of retinopathy in this and other selected studies**

| Severity of malaria | Retinal findings | Lewallen et al.^4^  (*n* = 735); children ^a,b^ | Beare et al.^6^  (*n* = 326);  children ^a,b^ | Kochar et al.^14^  (*n* = 424);  adults ^a,b^ | Looareesuwan  et al.^8^  (*n* = 144);  adults ^a^ | This study  (*n* = 66);  adults ^a,b,c^ |
| --- | --- | --- | --- | --- | --- | --- |
| Cerebral |  | (*n* = 439) | (*n* = 278) | (*n* = 214) | (*n* = 144) | (*n* = 20) |
|  | Any retinopathy |  | 61 | 34 |  | 70 |
|  | Haemorrhage | 39 | 46 | 9 | 15 | 55 |
|  | Papilloedema | 6 | 15 | 7 | 1 | 5 |
|  | Retinal whitening | 49 | 44/46 ^d^ | 2 | 4 | 45/50 ^d^ |
|  | Vessel discolouration | 20 | 32 | 0 | 0 | 0 |
| Severe non-cerebral |  | (*n* = 72) | (*n* = 47) ^e^ | (*n* = 58) |  | (*n* = 7) |
|  | Any retinopathy |  | 53 | 24 |  | 43 |
|  | Haemorrhage | 21 | 30 | 16 |  | 43 |
|  | Papilloedema | 1 | 4 | 21 |  | 14 |
|  | Retinal whitening | 26 | 45/23 ^d^ | 0 |  | 57/43 ^d^ |
|  | Vessel discolouration | 12 | 21 |  |  | 0 |
| Uncomplicated |  | (*n* = 141) ^f^ |  | (*n* = 152) |  | (*n* = 15) |
|  | Any retinopathy |  |  | 12 |  | 60 |
|  | Haemorrhage | 4 |  | 3 |  | 47 |
|  | Papilloedema | 0 |  | 1 |  | 0 |
|  | Retinal whitening | 6 |  | 0 |  | 53 |
|  | Vessel discolouration | 0 |  |  |  | 0 |

^a^ Used direct ophthalmoscopy.

^b^ Used indirect ophthalmoscopy.

^c^ Used retinal photography.

^d^ Shown as peripheral/macular whitening.

^e^ Severe malarial anaemia.

^f^ ‘Moderate’ plus ‘uncomplicated’ malaria.
